# The Efficacy of Single-Stage Correction by Posterior Approach for Neglected Congenital Scoliosis: Comparative Analysis According to the Age of Surgical Intervention

**DOI:** 10.3390/jcm11092278

**Published:** 2022-04-19

**Authors:** Jae Hyuk Yang, Hong Jin Kim, Dong-Gune Chang, Seung Woo Suh, Yunjin Nam, Jae-Young Hong

**Affiliations:** 1 Department of Orthopedic Surgery, Korea University Anam Hospital, College of Medicine, Korea University, Seoul 02841, Korea; kuspine@naver.com; 2 Department of Orthopedic Surgery, Inje University Sanggye Paik Hospital, College of Medicine, Inje University, Seoul 01757, Korea; hongjin0925@naver.com; 3 Department of Orthopedic Surgery, Korea University Guro Hospital, College of Medicine, Korea University, Seoul 08308, Korea; spine@korea.ac.kr (S.W.S.); nam.yunjin@gmail.com (Y.N.); 4 Department of Orthopedic Surgery, Korea University Ansan Hospital, College of Medicine, Korea University, Ansan 15355, Korea; osspine@korea.ac.kr

**Keywords:** congenital scoliosis, posterior approach, single-stage correction, osteotomy

## Abstract

Background: A single-stage correction for congenital scoliosis through a posterior-only approach is a commonly used surgical technique. However, there are few studies on the surgical treatment effect of posterior single-stage correction in patients with neglected congenital scoliosis. Methods: Patients who underwent a single-stage posterior correction for congenital scoliosis with a minimum follow-up of 2 years were divided into three groups based on age: Group A (7–11 years), B (12–18 years) and C (>18 years). A comparison of surgical, radiological, and clinical outcomes was performed for three groups. Results: The Cobb angle changed form 75 ± 18° to 37 ± 18° with a correction rate of 53%. Group A showed a significantly higher correction rate than Group B and C (all *p* < 0.001). The amount of blood loss in Groups B and C was significantly larger than that of Group A (*p* = 0.015). Pulmonary complications were significantly higher in Group C (*p* = 0.007). Conclusions: A single-stage correction with pedicle screws through a posterior-only approach achieved a significant correction with improved outcomes, even in neglected cases. However, the early correction for younger patients was still more beneficial in terms of bleeding loss, complications, and flexible curve correction.

## 1. Introduction

The treatment of congenital scoliosis focuses on early diagnosis and appropriate surgical management before the development of larger curves, since certain variants of congenital scoliosis have been known to progress more rapidly in comparison to other types of scoliosis [1,2]. Surgical treatment is usually recommended for patients with severe spinal deformity, but it is extremity challenging for the pediatric patients [1]. Early surgical intervention contributed to preventing the development of local deformities and secondary structural curves allowing normal growth in unaffected lesion of the spine [1,2,3,4,5].

Despite these recommendations, it is not uncommon to see cases of neglected congenital scoliosis, presenting in adulthood (after 18 years) [1,2]. There are several reasons for such a late presentation: a previously well-balanced deformity that was not exteriorly visible, as a result of ignorance on the part of care takers and, in some cases, due to fear of surgery [3]. It is known that, as time progresses, these curves become larger and stiffer rendering a surgical correction more challenging [4]. In addition, the deformation of costal bones frequently occurs causing secondary pulmonary insults such as restrictive lung disease and thoracic insufficiency [3]. Such larger and stiffer curves can theoretically make these patients more susceptible to higher chances of spinal cord injury during surgical interventions [4,5]. Late surgery can lead to the conducting of procedures, such as osteotomy and thoracoplasty, with associated risks [4,5,6]. In cases of severe congenital scoliosis, a combined anterior and posterior approach has been conventionally used, conducted in a sequential or staged manner to acquire flexibility by anterior release, followed by posterior corrective surgery [6,7]. In recent years, the three-column stability offered by pedicle screws allowed for a single-stage correction through a posterior-only approach [8,9,10].

However, at present, there is a dearth of research on the surgical outcome of the single-stage posterior-only approach in neglected congenital scoliosis (patients over 18 years of age). With this background, the objective of our study was to evaluate the surgical outcomes, including complication rates of single-stage posterior-only approaches to the correction of neglected congenital scoliosis. The study also aims to determine the age-related surgical efficacy of the posterior only approach for congenital scoliosis.

## 2. Materials and Methods

### 2.1. Study Design and Patients Group 

With the approval of an institutional review board, a retrospective chart review of all patients who underwent correction surgery for congenital scoliosis at our Scoliosis Research center from 2007 to 2012 was conducted. 

Among the enrolled patients, only patients who met the following conditions were primarily selected for this study: (1) Congenital scoliosis patients who were treated in a single-stage operation, (2) only a posterior surgical approach was used, and (3) minimum 2-year follow-up period. Secondarily, the following patients were excluded: (1) Revisional scoliosis surgery, (2) patients who may have had scoliosis due to other metabolic diseases, and (3) patients whose diagnosis could not be accurately identified.

The selected patients were divided into 3 groups based on the age at which surgical intervention was performed. Division of the three groups was based on the growth spurt at 11 years of age and the completion of growth at the age of 18. Group A with age range of 7–11 years, Group B with age range of 12–18 years, and group C (neglected congenital scoliosis group) included patients who were over 18 years old at time of surgical intervention. The surgical results of the neglected congenital scoliosis (Group C) group were evaluated in comparison to the two control groups (Group A and B).

For radiologic evaluation, whole-spine anterior–posterior (AP) and lateral radiographs were taken in all patients enrolled in this study. Through these radiographies, coronal alignment and sagittal balance were evaluated. Preoperative three-dimensional computed tomography (CT) was taken in all patients. Based on the CT results, the type of congenital scoliosis and the location of the congenital vertebrae were identified and a surgical plan was established based on these data of CT. Immediately after surgery, whole-spine AP and lateral radiographs were taken to confirm the status of the screw insertion and correction. Secondary radiographic imaging was performed 2 to 3 weeks after the operation, when the patient performed ambulation smoothly and could achieve a standing posture by themselves. To verify the maintenance of surgical correction, whole-spine AP and lateral radiographic images were taken at 3, 6 months, and 1 year after surgery and at the last follow-up.

### 2.2. Surgical Intervention

In this study, all enrolled patients underwent spinal correction while performing spinal cord neuromonitoring using a motor-evoked potential (MEP) device. The monitoring of the spinal cord using MEP was continued until the end of the operation.

The osteotomy was performed in accordance with Winter’s classification [11,12]. Single- or double-vertebral body resections, such as hemi- or block-vertebral body resections, were performed in the case of simple segmentation or formation defects by Winter’s classification. Smith-Peterson osteotomy and/or posterior multilevel crack osteotomy and/or vertebral column resection were performed with or without vertebral body resection in the case of coronal and sagittal imbalances of long segments with congenital vertebral anomaly [13]. A unilateral bar resection was also performed in the case of congenital scoliosis with unilateral bar. If the patients had a rib deformity and/or decreased flexibility of vertebral body, rib resection was also performed to obtain the flexibility of a vertebral body in the surgical process of unilateral bar resection. The 2-stage operation was performed in the case of a higher intraoperative bleeding, such as mixed-type bleeding, and/or intraoperative signal change of neuromonitoring systems, such as motor-evoked potential.

### 2.3. Measurements of Parameters

Hospital charts were reviewed for clinical details, per-operative findings and complications. For each group, the following factors were assessed: hospital stay, intensive care unit (ICU) admission, type of congenital scoliosis, extent of spine fusion, incidence of thoracoplasty, post-operative complications such as infection, pulmonary complications (hemothorax or pneumothorax), neurological deficit, re-operation and cerebro-spinal fluid (CSF) leakage.

Radiological analysis was conducted by a review of preoperative, postoperative (secondary post-operative radiography with standing posture), and last-follow-up radiographs. Coronal alignment was evaluated by Cobb angle, coronal balance (CB), T1 tilt angle (T1 angle), and T1 clavicle angle (CA). Cobb angle was used to calculate the postoperative correction rate (postoperative Cobb angle/preoperative Cobb angle × 100%). For coronal balance, left deviation from the central axis was marked as positive value, whereas right deviation was marked as negative value. For the CA and T1 angles, positive value was given for upper left area, and negative value was given for upper right area. When these values were compared to a reference value of 0, they were converted to an absolute value. In order to assess sagittal balance, sagittal vertical axis (SVA), thoracic kyphosis (TK), and lumbar lordosis (LL) were measured. SVA gave a positive value for anterior displacement and negative value for posterior displacement. This was converted to an absolute value when comparing it to the reference value of 0. All data of enrolled patients were described as median (range).

### 2.4. Statistical Analysis

Statistical analysis was performed using the SPSS program (version 18.0; IBM, Armonk, NY, USA); the Wilcoxon signed rank test and Kruskal–Wallis test were used for comparing mean values of continuous data, and the McNemar-Browker test and Fisher’s exact test was used to analyze categorical values. Post hoc analysis was performed by Bonferroni correction. *p*-values < 0.05 were considered statistically significant.

## 3. Results

**Figure 1:**
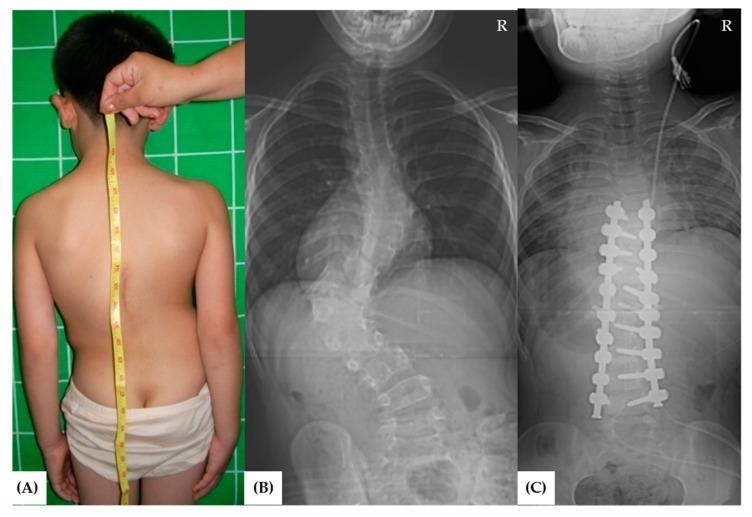
A 9-year-old boy patient (Group A) presented to the orthopedic clinic due to congenital scoliosis. (**A**) Clinical photograph. (**B**) the whole-spine anteroposterior view showed congenital scoliosis. The Cobb angle was 79°. (**C**) A hemi-vertebra resection and pedicle screw instrumentation by posterior approach were performed, and the curvature was corrected to 18° with a correction rate of 77% after the surgery. R: right-sided on radiographs.

**Figure 2:**
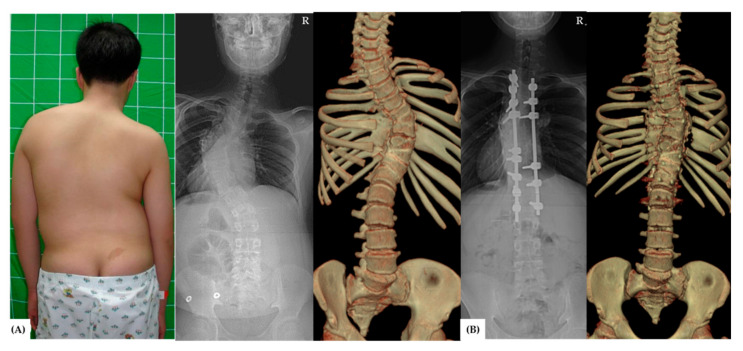
A 12-year-old adolescent patient (Group B) presented to the orthopedic clinic due to congenital scoliosis. (**A**) Clinical photograph: the whole-spine anteroposterior view, and three-dimensional computed tomography image showed congenital scoliosis. The Cobb angle was 81°. (**B**) Single-stage posterior correction with multiple crack osteotomies was performed, and the curvature was corrected to 29° with a correction rate of 67% after the surgery. R: right-sided on radiographs.

**Figure 3:**
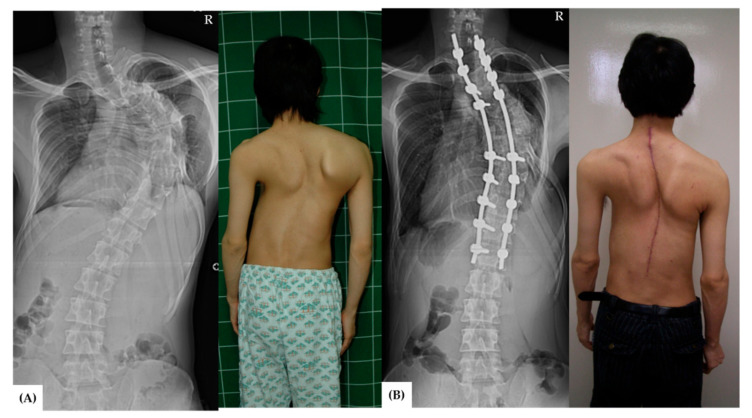
A 27-year-old male patient (Group C) presented to the orthopedic clinic due to neglected congenital scoliosis. (**A**) Clinical photograph and the whole-spine anteroposterior view showed congenital scoliosis. The Cobb angle was 89°. (**B**) Single-stage posterior correction by posterior vertebral column resection and thoracoplasty were performed, and the curvature was corrected to 40° with a correction rate of 55% after the surgery. R: right-sided on radiographs.

From 2007 to 2012, a total of 58 patients underwent congenital scoliosis surgery. Among them, a total of 37 patients met the inclusion and exclusion criteria for the study. Control groups of Group A (Figure 1) and B (Figure 2) included 11 and 17 patients, respectively, and experimental group C (Figure 3) included 9 patients.

As there was no statistically significant difference in the gender ratio of the group, type of congenital scoliosis, preoperative Cobb angle and follow-up period, the comparison between the three groups was made assuming that there were no differences between them. All cases in Group C required vertebral body osteotomy, with 5 cases (55%) needing thoracoplasty (Table 1).

The preoperative Cobb angle was 75° (50–104°), and postoperatively, it was 37° (15–75°), which shows a correction rate of 53% (28–71%). In case of control groups, the Cobb angle changed from 66° (10–152°) to 15° (2–63°) and 64° (21–130°) to 27° (2–56°), in Group A and Group B, respectively. With the correction rates being 77% (55–98%) for Group A and 57% (16–100%) for Group B. The correction rate showed a statistically significant difference between the three groups (*p* = 0.006). In the post hoc analysis, the comparisons between group A and B, group A and C were significant (*p* = 0.024, and *p* = 0.01), but group B and C were not significant. The values of coronal factors did not show significant differences between the three groups (Table 2).

Sagittal factors were as described in Table 3. On average, the SVA of Group C was corrected by 3 mm. TK was corrected from 43° (2–89°) to 35° (6–62°) and the LL from 34° (−14–73°) to 29° (−11–67°). Sagittal factors between the groups did not show statistically significant differences (Table 3).

The operation time, intraoperative blood loss, length of hospitalization, ICU admission, and complications in enrolled patients were recorded and are shown in Table 4. The surgical time of Group B and C was relatively longer than that of Group A; however, it was not statistically significant (*p* = 0.111). The amount of blood loss in Group B and C was larger than that of Group A, where the difference was statistically significant (*p* = 0.015). Regarding the complications, Group C showed a higher complication rate (88.9%) than Group A (18.2%) and group B (17.6%) with a statistical significance (*p* < 0.005). The occurrence of pulmonary complications for pneumothorax were higher in Group C. Although not statistically significant, other complications occurred more frequently in Group C (Table 4).

## 4. Discussion

The treatment of congenital scoliosis is focused on early diagnosis and proper surgical intervention before the development of severe deformity [1,2,3]. Some studies reported on surgical outcomes by age at the time of surgery [13,14]. However, no studies were conducted to compare the efficacy regarding the benefits and risks of a single-stage correction by a posterior approach in neglected congenital scoliosis. From our study, the surgical intervention of the patients with neglected congenital scoliosis showed a comparable correction rate but still had risks in terms of blood loss, complication rate, and difficulties in surgical correction.

The age of intervention is one of the most crucial factors in the management of congenital scoliosis [14,15]. This is due to the fact that it is better to stabilize a curve when it is small, and prevent it from worsening, than to correct the deformity when the child is grown up, when the curve becomes larger and stiffer [1,12]. Additionally, curves of congenital scoliosis are characterized by a rapid progression during first five years of life and at a pubertal growth spurt [16]. Hence, for certain variants of congenital scoliosis, the surgical approach varies based on the time of intervention along the natural course of the curves [2]. For example, for patients younger than 5 years with congenital scoliosis, it is known that fusion can have deleterious effect on thoracic volume [17]. Hence, in the absence of rib fusions, the growing rod technique has replaced spinal arthrodesis as the standard of care in this age group [18]. However, instrumentation and fusion at the earliest possible age are shown to have favorable results in congenital scoliosis patients presenting in juvenile and adolescent age groups [8,9,14]. In recent years, a single-stage posterior pedicle screw-based approach has become well-established as a safe and effective method in this age group [9,10]. Nevertheless, the outcomes of surgical intervention in adults with neglected congenital scoliosis (>18 years) are yet to be elucidated. 

There is paucity of literature on single-stage posterior corrections for neglected congenital scoliosis. Recently, Sarlak et al. reported an isolated posterior approach in 14 congenital scoliosis patients with a mean age of 14.9 years at surgery (range: 10 to 25 years) [10]. In the study by Sarlak et al., the deformities were mainly corrected by the compression of the convex deformity side with the segmental resection of three apical ribs after pedicle screw instrumentation without any usage of osteotomy techniques, which was able to achieve a 51.6% correction rate [10]. In a more recent study, Ayvaz M et al. reported the results of single-stage posterior correction in eighteen adolescent congenital kyphoscoliosis patients with a mean age of 13.6 years (range, 11–16 years) [19]. Chevron osteotomies were performed at apical segments (three to seven levels) with concave rib osteotomies, resulting in a correction rate of 62% [19]. 

Our Cobb angle correction rates in Group B and Group C were similar to the results of the above studies [10,19]. In our study in Group C (the neglected congenital scoliosis group), the Cobb angle was corrected by 39° (range, 26–53°) with a correction rate of 53% (range, 28–71%), which was a similar to correction in the adolescent group, Group B. However, in our research, we also found that in patients younger than 11 years—denoted as the juvenile group, Group A—the correction rate was significantly higher than that of the adult and adolescent groups, Groups B and C (77% vs. 57% and 53%). In accordance with these results, it can be stated that, for similar Cobb angles, an earlier surgical intervention can result in a more effective correction than delayed intervention. It is possible that this difference is due to the curves becoming more rigid with age, which coincides with other studies [9,10,13,14]. Additionally, the concomitant progression of the deformation of the rib cage can add to the rigidity and the magnitude of the curve. The correction rates in Group C, point to the fact that, even though the skeletal curves become stiff with age, they can still yield to posterior-only approaches owing to the three-column stability of pedicle screws and release versatility of the available osteotomies [9]. 

Though not statistically significant the surgical time in Group A was shorter than in Group B and Group C. Additionally, the average intraoperative blood loss in Group A was less than that in Group B and Group C. This difference in average blood loss between Group A and Group B and C was statistically significant (*p* = 0.015). The following factors could be attributed to the relatively decreased blood loss: Firstly, due to their relatively young age, Group A patients required less additional osteotomy or costectomy than in Group B and C, owing to more flexible vertebrae, except at the site of deformation. Secondly, the amount of soft-tissue detachment for the approach to osteotomy or costectomy could have been smaller, obtaining flexibility during the posterior approach of vertebral body detachment. In the study by Ayvaz M et al. on neglected adolescent congenital scoliosis, the authors reported similar results at a surgical time of 292 min with average bleeding of 989 mL [19]. In a similar adolescent age group, Sarlak et al. also reported similar results for a mean operation time of 3.5 h (210 min) and a mean blood loss of 980 mL (range: 450 to 2200 mL) [10].

The incidence of surgical complications was significantly higher in Group C compared to Group A and B. The authors consider that the following are reasons for the high incidence of surgical complications in Group C: Various difficult surgical techniques are needed in the cases of neglected congenital scoliosis (Group C), which is reflected on the result of complication rate [9]. In patients with neglected congenital scoliosis (Group C), rib deformity and spontaneous fusion between the rips around the deformed spine were often accompanied by congenital spinal deformity, and the flexibility of the vertebral body was often poor. Therefore, there were many cases of rib resection in order to obtain an appropriate correction angle during corrective spine surgery (rib resection rates 9%, 41% and 55% in Groups A, B and C) [19]. Due to these reasons, the incidence of pulmonary complications such as pneumothorax and hemothorax was higher in Group C. Ayvaz et al. also reported that adolescent congenital scoliosis patients underwent rib resections during surgery and reported pulmonary complications after surgery [19]. Furthermore, in younger groups, severe thoracic deformity limits lung capacity, for which deformity correction improved pulmonary function. The improvement of thoracic cage after deformity correction permits the growth of the lungs during development [9]. However, in mature adult groups, all were fully grown, which called for more difficult surgical techniques, and more pulmonary complications appeared in limited lung capacity [9,14]. 

The incidence of neurological deficits in Group C was relatively higher than in Groups A and B (9%, 0%, 22% in Group A, B and C), but did not reach statistically significance (*p* = 0.083). We speculate that, in the process of correction of the spine, an uncontrolled traction force was transmitted to the spinal cord and dynamic instability and venous engorgement or arterial stretching around corrected deformed spine can occur. All these situations might have caused injury [20]. The risk of such injury was higher in Group C due to a decrease in the flexibility of aged spines, and the progressive deformation of vertebral body caused by neglect [17]. Rajavelu et al. reported that, when performing surgery to correct neglected congenital spinal deformity—if there is a kyphotic or kyphoscoliotic deformity in the congenitally deformed vertebra, a formational defect and mixed type of vertebral anomalies, and proximal thoracic vertebral lesions—there was a high probability of developing a neurological deficit [20]. In this study, type 1 and 3 other types of spinal deformity were relatively common in Group C (67%, 45% in Group A, 29% in Group C). The lesser complication rates in Group A of our study population were mirrored in previously published reports on the posterior correction of congenital scoliosis [21,22,23].

From our study, the surgical correction of neglected congenital scoliosis had acceptable outcomes regardless of age at surgery. However, delayed surgery in congenital scoliosis made it difficult for deformity correction due to an increased rigidity of the vertebral body and the necessity of multiple and various types of osteotomy, which showed increased postoperative complications [24,25]. Furthermore, Group C showed a higher fusion level, intraoperative bleeding, and hospital stay than Group A and B, indicating that the risk of surgery was higher in Group C. Therefore, even if single-stage correction by a posterior approach also obtained a comparable correction rate, early surgery was important for reducing complication rates because the case of neglected congenital scoliosis requires extensive surgical procedures.

Our study is limited by its retrospective nature and the small number of patients, who could not be categorized based on the type of congenital scoliosis and various surgical techniques [26]. Additionally, some comparisons that showed no significant statistical significances may be due to our small sample size. However, considering the paucity of neglected congenital scoliosis patients, gathering the patient groups by homogeneity of age and type of deformity is very difficult. Additionally, no clinical satisfaction measure was assessed to study the impact of intervention on the quality of life of the patients as there is no scale or index which has been validated for the measurement of clinical outcomes in congenital scoliosis. In spite of these limitations, this study is still relevant as it enabled us to ascertain the effectiveness of a posterior-only approach for adult neglected congenital scoliosis and demonstrated the differences in outcomes of congenital scoliosis based on the age of surgical intervention.

## 5. Conclusions

A single-stage correction with pedicle screws through a posterior-only approach achieves a significant correction with improved outcomes, even in neglected cases. However, the early correction for younger age patients is still more beneficial in terms of bleeding loss, complications rate, and flexible curve correction.

## Figures and Tables

**Table 1:** Demographics of enrolled patients.

Factors	Group A (7–11 Years)	Group B (12–18 Years)	Group C (Age > 18 Years)	*p* Value
Age (years)	8.8 (7–11)	13.8 (12–15)	27.3 (18–15)	<0.001
Sex (male/female)	6/5	9/8	6/3	0.834
Follow-up period (month)	95.4 (53–131)	91.3 (53–137)	93.4 (60–146)	0.998
Pre-op Cobb angle (°)	66 (10–152)	64 (21–130)	75 (50–104)	0.259
Congenital scoliosis type (1/2/3)	3/6/2	3/12/2	3/3/3	0.433
Congenital vertebrae resection (hemi or block vertebral body resection)	7	8	7	0.012
Osteotomy except congenital vertebrae resection	1	7	9	-
Additional osteotomy	4	9	7	0.180
Correction without osteotomy	1	2	0	-
Thoracoplasty (yes/no)	1/10	7/11	5/4	0.238

Group A, B, C are neglected congenital scoliosis aged between 7 to 11 years, congenital scoliosis aged between 12 and 18 years, and neglected congenital scoliosis aged over 18, respectively. Congenital scoliosis type was differentiated to the defect of vertebral segmentation (type 1), defects of vertebral body formation (type 2) and mixed anomalies (type 3). It was also described in the same order as in this table. Additional osteotomies used for correction of spinal deformity are Smith-Peterson, Ponte, multiple crack osteotomy and vertebral column resection. Significant differences are accepted for *p* < 0.05.

**Table 2:** Coronal factors of enrolled patients.

Factors	Group A (7–11 Years)	Group B (12–18 Years)	Group C (Age > 18 Years)	*p* Value
Pre-Op Cobb angle (°)	66 (10–152)	64 (21–130)	75 (50–104)	-
Post-Op Cobb angle (°)	15 (2–63)	27 (2–56)	37 (15–75)	-
∆ Cobb angle	51 (15–100)	38 (7–130)	39 (26–53)	0.970
Statistical significance * (Cobb angle)	0.003	<0.001	0.008	-
Correction rate ^$^ (%)	77 (55–98)	57 (16–100)	53 (28–71)	0.006
Pre-Op Coronal balance (mm)	1 (−25–44)	−1 (−65–59)	6 (−17–50)	-
Post-Op Coronal balance (mm)	6 (−15–37)	−4 (−38–36)	9 (−32–41)	-
∆ Coronal balance (mm)	5 (−3–17)	9 (−31–65)	1 (−19–11)	0.348
Statistical significance * (Coronal balance)	0.575	0.438	0.678	
Pre-Op T1 tilt angle (°)	0 (−15–23)	−5 (−30–33)	−2 (−26–33)	
Post-Op T1 tilt angle (°)	0 (−15–9)	−1 (−14–22)	1 (−14–16)	
∆ T1 tilt angle (°)	2 (−11–14)	7 (−17–28)	4 (−5–17)	0.472
Statistical significance * (T1 tilt angle)	1.00	0.271	0.173	
Pre-Op T1 clavicle angle (°)	0 (−9–7)	−1 (−14–9)	−2 (−6–0)	
Post-Op T1 clavicle angle (°)	0 (−12–9)	0 (−9–8)	−1 (−5–2)	
∆ T1 clavicle angle (°)	0 (−8–4)	1 (−7–6)	1 (−4–4)	0.595
Statistical significance * (T1 clavicle angle)	0.477	0.232	0.161	

Group A, B, C are neglected congenital scoliosis aged between 7 to 11 years, congenital scoliosis aged between 12 and 18 years, and neglected congenital scoliosis aged over 18, respectively. Statistical significance * was statistically analyzed between preoperative and postoperative variables. ^$^ Post hoc analysis of different groups by Bonferroni test at 95% confidence level. Group A vs. Group B: *p* = 0.024, Group A vs. Group C: *p* = 0.010, and Group B vs. Group C: *p* = 1.00.

**Table 3:** Sagittal factors of enrolled patients.

Factors	Group A (7–11 Years)	Group B (12–18 Years)	Group C (Age > 18 Years)	*p* Value
Pre-Op SVA (mm)	7 (−46–105)	5 (−60–124)	27 (−27–117)	
Post-Op SVA (mm)	26 (−2–70)	15 (−66–104)	37 (−30–182)	
∆ SVA (mm)	3 (−30–39)	9 (−68–104)	3 (−65–78)	0.910
Statistical significance * (SVA)	0.169	0.196	0.678	
Pre-Op TK (°)	55 (15–119)	28 (3–70)	43 (2–89)	0.094
Post-Op TK ^$^ (°)	41 (15–105)	20 (3–49)	35 (6–62)	0.020
Statistical significance * (TK)	0.168	0.017	0.477	
Pre-Op LL (°)	49 (2–75)	47 (−26–100)	34 (14–73)	0.5
Post-Op LL (°)	46 (28–75)	42 (0–72)	29 (11–67)	0.303
Statistical significance * (LL)	0.790	0.218	0.859	

Group A, B, C are neglected congenital scoliosis aged between 7 to 11 years, congenital scoliosis aged between 12 and 18 years, and neglected congenital scoliosis aged over 18, respectively. Statistical significance * was statistically analyzed between preoperative and postoperative variables. ^$^ Post hoc analysis of different groups by Bonferroni test at 95% confidence level. Group A vs. Group B: *p* = 0.028, Group A vs. Group C: *p* = 1.00, and Group B vs. Group C: *p* = 0.161. SVA = sagittal vertical axis; TK = thoracic kyphosis; LL = lumbar lordosis.

**Table 4:** Operative factors and complications of enrolled patients.

Factors	Group A (7–11 Years)	Group B (12–18 Years)	Group C (Age > 18 Years)	*p* Value
Operation time (min)	229 (100–386)	326 (152–710)	316 (198–463)	0.111
Fusion extent	7.8 (1–13)	9.0 (1–16)	8.8 (2–15)	0.482
Bleeding loss ^$^ (mL)	1564 (300–4000)	3271 (700–6000)	3644 (800–8000)	0.015
Hospital stay (day)	20 (11–47)	20 (12–61)	34 (13–141)	0.337
ICU stay (yes/no)	1/10	3/14	1/8	1.00
Complications	2	3	8	<0.005
Hemothorax	0	1	1	0.211
Pneumothorax	0	2	5	0.003
Infection	1	0	0	1.00
Neurologic deficit	1	0	2	0.083
CSF leakage	0	0	0	-

Group A, B, C are neglected congenital scoliosis aged between 7 to 11 years, congenital scoliosis aged between 12 and 18 years, and neglected congenital scoliosis aged over 18, respectively. ^$^ Post hoc analysis of different groups by Bonferroni test at 95% confidence level. Group A vs. Group B: *p* = 0.025, Group A vs. Group C: *p* = 0.046, and Group B vs. Group C: *p* = 1.00. ICU, intensive care unit; CSF, cerebro-spinal fluid.

## Data Availability

The data collected for this study, including individual patient data, will not be made available.
